# Molecular and Paleontological Evidence for a Post-Cretaceous Origin of Rodents

**DOI:** 10.1371/journal.pone.0046445

**Published:** 2012-10-05

**Authors:** Shaoyuan Wu, Wenyu Wu, Fuchun Zhang, Jie Ye, Xijun Ni, Jimin Sun, Scott V. Edwards, Jin Meng, Chris L. Organ

**Affiliations:** 1 Department of Organismic and Evolutionary Biology and Museum of Comparative Zoology, Harvard University, Cambridge, Massachusetts, United States of America; 2 Institute of Vertebrate Paleontology and Paleoanthropology, Chinese Academy of Sciences, Beijing, China; 3 Xinjiang Key Laboratory of Biological Resources and Genetic Engineering, Xinjiang University, Urumqi, Xinjiang, China; 4 Key Lab of Cenozoic Geology and Environment, Institute of Geology and Geophysics, Chinese Academy of Sciences, Beijing, China; 5 Division of Paleontology, American Museum of Natural History, New York, New York, United States of America; Monash University, Australia

## Abstract

The timing of the origin and diversification of rodents remains controversial, due to conflicting results from molecular clocks and paleontological data. The fossil record tends to support an early Cenozoic origin of crown-group rodents. In contrast, most molecular studies place the origin and initial diversification of crown-Rodentia deep in the Cretaceous, although some molecular analyses have recovered estimated divergence times that are more compatible with the fossil record. Here we attempt to resolve this conflict by carrying out a molecular clock investigation based on a nine-gene sequence dataset and a novel set of seven fossil constraints, including two new rodent records (the earliest known representatives of Cardiocraniinae and Dipodinae). Our results indicate that rodents originated around 61.7–62.4 Ma, shortly after the Cretaceous/Paleogene (K/Pg) boundary, and diversified at the intraordinal level around 57.7–58.9 Ma. These estimates are broadly consistent with the paleontological record, but challenge previous molecular studies that place the origin and early diversification of rodents in the Cretaceous. This study demonstrates that, with reliable fossil constraints, the incompatibility between paleontological and molecular estimates of rodent divergence times can be eliminated using currently available tools and genetic markers. Similar conflicts between molecular and paleontological evidence bedevil attempts to establish the origination times of other placental groups. The example of the present study suggests that more reliable fossil calibration points may represent the key to resolving these controversies.

## Introduction

Molecular clocks and fossil record are the two major approaches to date evolutionary divergence times, which are crucial for using the Tree of Life to understand evolutionary processes and mechanisms. In the case of major divergences among groups of placental mammals, the general tendency has been for paleontological studies to suggest that these events took place in the Paleocene, while molecular ones place them deep in the Cretaceous [1], [2], although some molecular studies have recovered estimated divergence times that are more compatible with the fossil record [3], [4], [5], [6]. The general pattern of disagreement has confounded our ability to discern the influence of the K/Pg extinction event on the radiation of extant mammals. This problem is particularly evident in rodents, a group that accounts for approximate 42% of extant mammalian diversity [7]. The oldest known fossil that can be clearly identified as a member of the rodent lineage, the fragmentary possible crown-rodent *Acritoparamys*, has a Late Paleocene age of about 57 million years (Ma) [8], [9]. However, early molecular studies almost unanimously supported a Cretaceous radiation of rodents [5], [6], [10], [11], [12], [13], [14], [15], [16], although Douzery et al. [3] and Kitazoe et al. [4] obtained molecular results that placed the earliest divergences within crown-Rodentia in the Paleocene and was therefore compatible with the fossil record. Despite these exceptions, a strong discrepancy still persists between the fossil record and the preponderance of results from molecular clock studies. Resolving this discrepancy is therefore critical not only for understanding the evolutionary history and dynamics of rodents, but also for assessing the reliability of molecular clocks and fossils to accurately estimate divergence times.

Molecular clocks attempt to pinpoint divergence events whereas the fossil record alone can yield minimum estimates given by the first known fossil occurrence for a given group [1], [2]. The problem of molecular rate heterogeneity, a major source undermining the accuracy of molecular clock estimates, has been addressed by applying relaxed molecular clocks across sequences [17]. The availability of reliable fossils that can be used as calibration points, therefore, may hold the key to obtaining an accurate time estimate of the origin and radiation of rodents. In this study, we employed five rodent calibrations based on recent fossil discoveries, including two new fossils that are chronologically constrained with palaeomagnetic chrons. The earliest known fossils of Dipodinae (three-toed jerboas) is dated to 10.5 Ma from the beginning of the Late Miocene of China (Fig. S1 and Fig. S2), providing an upper (more recent) bound for the divergence time between Dipodinae and Allactaginae (five-toed jerboas). The earliest known fossils of the two extant genera of Cardiocraniinae (dwarf jerboas), *Cardiocranius* and *Salpingotus*, are dated to 9 Ma [18]. The lower (more ancient) bound of each of these divergence events is 13 Ma, in the middle part of the Middle Miocene, based on recent biostratigraphic and paleomagnetic data. The earliest known myodont, *Erlianomys*, was discovered from the Early Eocene (54 Ma) [19], providing a reliable lowest known bound for the divergence time between the two primary myodont groups, Dipodoidea and Muroidea (Fig. S3) [20]. The upper bound of this divergence can be constrained to 43 Ma, based on the earliest known dipodoids [21] and muroids [22] from China. We therefore use the split between mice and rats [23] as well as between octodontids and erethizontids [24], [25] as calibration points, because of their improved fossil record and stratigraphic data. In order to achieve a balanced distribution of calibration points within the phylogeny, we also applied two well-defined non-rodent calibrations in successive sister lineages to rodents, including the split of marsupials and placentals [26], [27], and feliforms and caniforms [28].

We use the fossils noted above to create seven fossil calibrations with a nine-gene sequence dataset to re-evaluate the timing of rodent origin and diversification. For taxon sampling, we included major lineage across rodents, and sampled comprehensively within Dipodoidea to include all six subfamilies, an approach we referred to as “bottom-up” taxon sampling (i.e. building up an analytic model from a foundation of many individual data samples, versus the “top-down” approach of inferring an analytic model from relatively few data points). This sampling approach allowed us to accurately incorporate these new calibration points based on Chinese dipodoid fossils that are comparatively recent in geological time. Our analysis implements a relaxed molecular clock model using Bayesian and maximum likelihood approaches. Our results suggest that rodents originated and diversified after the K/Pg boundary at the beginning of the Cenozoic, a finding consistent with patterns found in the fossil record.

## Results

### Test of molecular rate heterogeneity

The BEAST [29] analysis shows that substantial rate variation across the data set was only found in the CNR1 locus, with an ucld.stdev parameter of 1.788 (95% confidence interval (CI) 1.35–2.19). The ucld.stdev values of all other loci lie between 0 and 1, indicating that these loci have a moderate level of rate variation (see Table S1). The ucld.stdev parameter of the concatenated partitioned data set is 0.57 (95% CI 0.45–0.71), which is lower than that of all other individual genes except IRBP. These results demonstrate the necessity of utilizing a relaxed molecular clock mode on the DNA dataset for the molecular dating estimates.

### Molecular dating with complete fossil constraints

**Figure 1:**
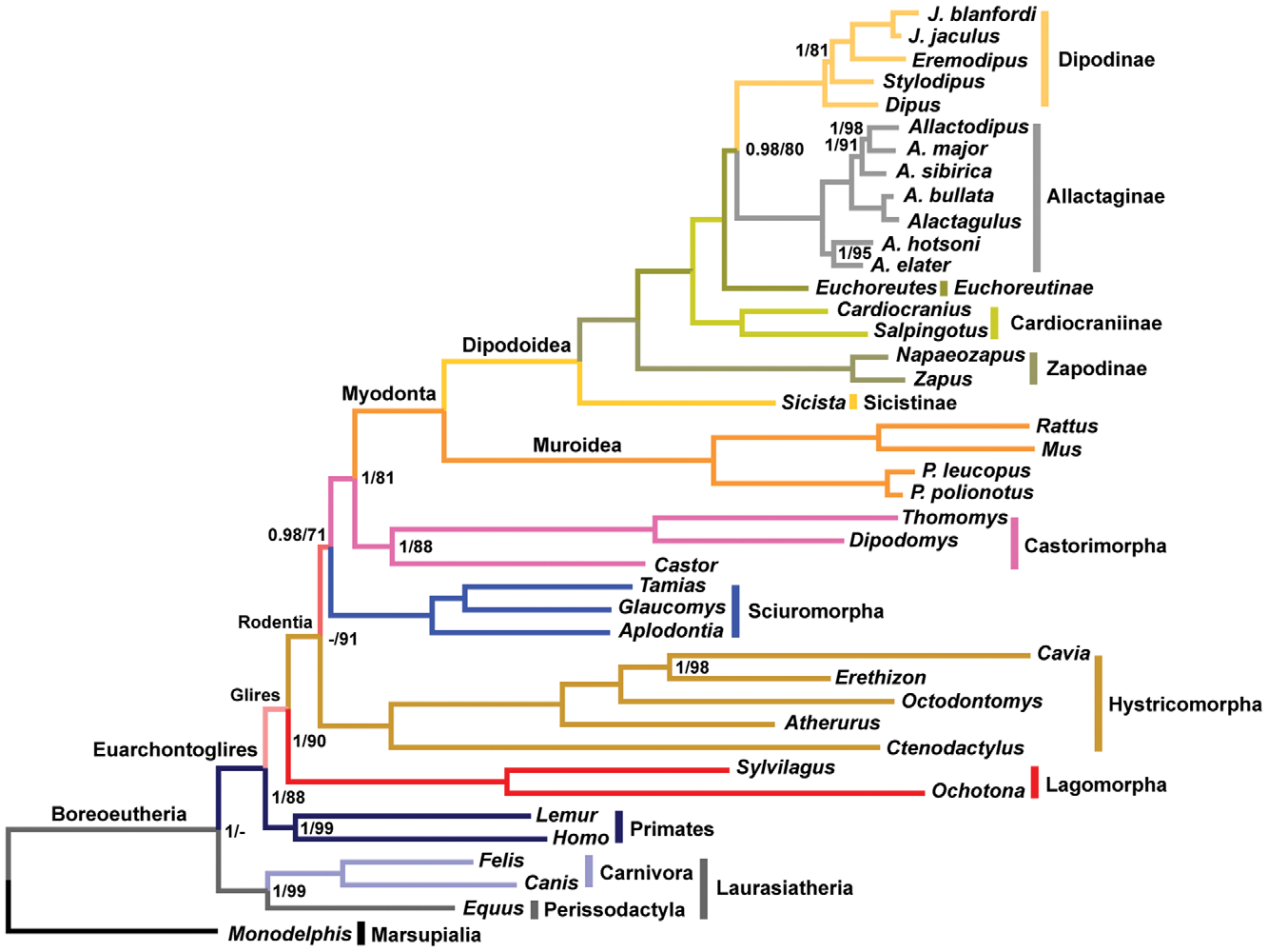
Phylogenetic relationships for rodents and outgroups estimated using Bayesian and maximum likelihood algorithms. The Bayesian posterior probability and the maximum likelihood bootstrap values for each of the nodes are provided from the left to the right of the slash, respectively. Support scores are not shown for nodes that receive a full support of both posterior probability and bootstrap value.

**Figure 2:**
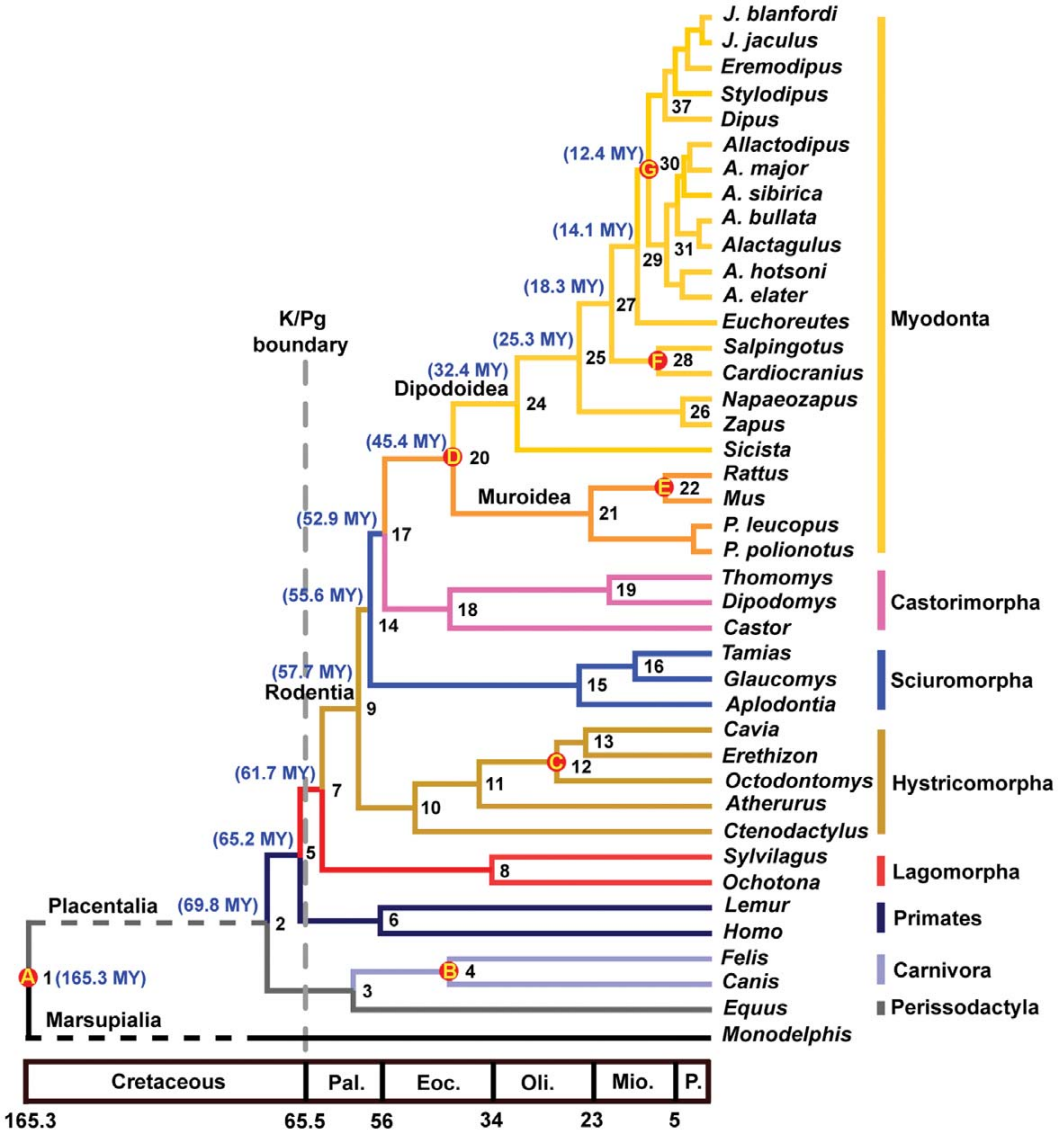
Molecular time scale for the orders of Rodentia, Lagomorpha, Primates, Carnivora and Perissodactyla obtained from the Bayesian estimates based on seven fossil calibration points and relaxed molecular clock model. Fossil constraints are indicated by circle A to G on the corresponding nodes: **A**. 160–190 Ma for the split between Placentalia and Marsupialia; **B**. 38–61.7 Ma for the split between Caniformia and Feliformia; **C**. 28.5–37 Ma for the split between *Octodontomys* and *Erethizon*; **D**. 43–54 Ma for the basal split of Myodonta; **E**. 7.3–12.2 Ma for the split between mice and rats; **F**. 9–13 Ma for the split between *Cardiocranius* and *Salpingotus*; **G**. 10.5–13 Ma for the split between Dipodinae and Allactaginae.

We inferred a time-calibrated phylogenetic tree for 41 mammal species focusing on the superfamily Dipodoidea (jerboas and relatives) for which we sampled 18 species, representing all six subfamilies. Nine unlinked nuclear genes were used to construct the tree using Bayesian [30] and likelihood [31] criteria. Topologically, the Bayesian and maximum likelihood approaches gave identical, highly resolved phylogeny, supporting Hystricomorpha as the most basal rodent clade (bootstrap support = 71, posterior probability = 0.98, Fig. 1). For time-calibrated trees, we estimated divergence times using Bayesian [29] (Fig. 2) and likelihood [32] approaches, which returned similar results (Table 1). Our estimated divergence times are much younger than those estimated in previous molecular analyses, and are more congruent with the fossil record.

**Table 1:** Support values of divergence dates for Bayesian and penalized likelihood estimates using seven fossil calibration points.

Nodes	Description of Nodes	Bayesian estimates	Foss. Cons. Exp. Dist.				Likelihood estimates	Uniform constraint	
			ESS	Mean	offset	95% cre. int.		Mini.	Maxi.
1	Marsupialia-Placentalia	165.3 (160–175.7)	8861	3.9	124.6	160–190	-	160	160
2	Base of Boreoeutheria	69.8 (58.6–81.9)	257	-	-	-	69.71	-	-
3	Perissodactyla-Carnivora	56.5 (44.2–69.5)	691	2.5	62.3	62.36–71.52	60.7	-	-
4	Caniformia-Feliformia	41.6 (38–48.4)	1992	6.5	38	38–61.98	42.55	38	61.7
5	Primates-Glires	65.2 (55–75.4)	254	-	-	-	65.35	-	-
6	Hominoidea-Lemuroidea	55.6 (40.4–70)	697	-	-	-	57.86	-	-
7	Lagomorpha-Rodentia	61.7 (52.8–71)	251	-	-	-	62.37	-	-
8	Ochotonidae-Leporidae	34.6 (18.8–50.7)	386	-	-	-	38.11	-	-
9	Base of Rodentia	57.7 (50.1–66)	245	-	-	-	58.89	-	-
10	Base of Hystricomorpha	50.2 (41.6–58.7)	445	-	-	-	52.32	-	-
11	Hystricidae-Caviomorpha	36.9 (31.6–43)	1095	-	-	-	35.19	-	-
12	*Octodontomys-Erethizon*	29.8 (28.5–32.2)	4214	2.3	28.5	28.56–36.98	28.5	28.5	37
13	*Cavia-Erethizon*	24.6 (17.6–30)	876	-	-	-	25.52	-	-
14	Myodonta-Sciuromorpha	55.6 (48.4–63.2)	251	-	-	-	56.98	-	-
15	Base of Sciuromorpha	25.4 (11.9–38.6)	245	-	-	-	35.87	-	-
16	*Glaucomys-Tamias*	17.8 (7.3–29.9)	228	-	-	-	27.54	-	-
17	Myodonta-Castorimorpha	52.9 (46.5–59.9)	268	-	-	-	54.57	-	-
18	Base of Castorimorpha	45.5 (33.9–55.3)	408	-	-	-	49.96	-	-
19	Heteromyidae-Geomyidae	20.6 (9.9–32.2)	453	-	-	-	22.31	-	-
20	Muroidea-Dipodoidea	45.4 (43–49.4)	539	3	43	43.08–54.07	46.08	43	54
21	Cricetidae-Muridae	23.4 (14.7–32.2)	561	-	-	-	20.93	-	-
22	Mouse-Rat	9.5 (7.3–12.9)	1206	1.4	7.3	7.335–12.46	10.96	7.3	12.2
24	Base of Dipodoidea	32.4 (25.2–39.7)	515	-	-	-	28.85	-	-
25	Zapodidae-Dipodidae	25.3 (19.1–31.7)	528	-	-	-	22.47	-	-
26	*Zapus-Napaeozapus*	5.7 (1.9–10.1)	1063	-	-	-	3.5	-	-
27	Base of Dipodidae	18.3 (13.9–22.8)	664	-	-	-	16.15	-	-
28	*Salpingotus-Cardiocranius*	10.2 (9–12.4)	4295	1.1	9	9.028–13.06	11.27	9	13
29	Euchoreutinae-Allactaginae	14.1 (11.2–17.3)	693	-	-	-	12.24	-	-
30	Dipodinae-Allactaginae	12.4 (10.5–14.9)	747	0.7	10.5	10.52–13.08	11.29	10.5	13
31	Base of Allactaginae	7.7 (5.4–9.9)	562	-	-	-	4.53	-	-
37	Base of Dipodinae	7.5 (5–9.8)	627	-	-	-	4.25	-	-

Values in parentheses are the 95% credibility intervals. - indicates that the corresponding node was not present in the corresponding analyses. Abbreviations: Foss. Cons. Exp. Dist., fossil constraints set as exponential distribution; ESS, effective sample size; Cre. Int., credibility interval; Mini, minimum; Maxi, Maximum.

We estimate that the divergence between rodents and lagomorphs occurred about 61.7 Ma (Bayesian, Bayesian credibility interval (BCI) 52.8–71) or 62.4 Ma (likelihood). The basal divergence of rodents was estimated to be 57.7 Ma (Bayesian, BCI 50.1–66) or 58.9 Ma (likelihood). These estimates are roughly consistent with recent interpretations of the early gliran fossil record. The oldest known Glires, *Heomys* and *Mimotona*, may both fall on the lagomorph stem [33], although Meng et al. [9] recovered *Heomys* as a stem-rodent rather than a stem-lagomorph. However, both analyses agree that *Heomys* and *Mimotona* belong to the gliran lineage. Both genera are placed in the early Late Paleocene [9], [34], an age close to the rodent-lagomorph divergence time estimated by our study. The appearance of sciuromorphs was estimated at 55.6 Ma (Bayesian, BCI 48.4–63.2) or 57 Ma (likelihood). The divergence between castorimorphs and myodonts was estimated at 52.9 Ma (Bayesian, BCI 46.5–59.9) or 54.6 Ma (likelihood). In addition, we estimate the basal divergence of hystricomorphs to be 50.2 Ma (Bayesian, BCI 41.6–58.7) or 52.3 Ma (likelihood), a result consistent with the oldest hystricomorph fossils, which date to ∼50 Ma [35]. We estimate the origin of crown Hystricognathi to be 36.9 Ma (Bayesian, BCI 31.6–43) or 35.2 Ma (likelihood), compatible with the occurrence of the oldest hystricid fossils at ∼34 Ma [36].

We tested for the Node Density Effect (NDE) [37], [38] using the online utility available at (http://www.evolution.reading.ac.uk/pe/index.html), which is based on the method outlined by Venditti et al. (2006) [39]. The results of this test indicate that the NDE is present in our tree (β significantly greater than zero). By successively pruning clades in the tree, we find that the NDE is present because of the correlation between nodes and branch lengths associated with the increasing sampling of Dipodinae and Allactaginae in relation to the rest of the tree. Reducing the numbers of taxa within Dipodinae and Allactaginae or pruning out Dipodinae removes the NDE. The NDE may have the effect of causing our estimates of divergence time to appear more recent, because unsampled lineages could downwardly bias the molecular divergences. To test this, we re-analyzed our data set using BEAST with reduced taxon sampling in the subfamilies Dipodinae and Allactaginae. For these two subfamilies, we sampled two taxa for each, including *Dipus sagitta* and *Jaculus blanfordi* for Dipodinae, and *Allactaga elater* and *Allactodipus bobrinskii* for Allactaginae, because such taxon sampling removes the NDE from our tree. Our results show that the BEAST analysis based on the reduced tree produced divergence times for major nodes that are similar to those for the full taxa (Table S6), and statistically we found no significant difference between these two estimates (t-test, p-value = 0.954). These results suggest that the impact of the NDE on the estimated divergence times for the rest of the tree is limited.

Although the confidence intervals attached to our Bayesian estimates are relatively wide, as is often the cases for studies like this one that employ a limited number of genes [6], the fact that our Bayesian and maximum likelihood estimates generally agree with one another reinforces the results of both analyses and suggests that the dates are reliable.

### Sensitivity test of fossil age constraints

We tested the sensitivity of applying different fossil constraints for their impact on the estimated times of divergence within rodents.

### Bayesian

Compared with the estimated dates using all constraints, omitting constraint D for the divergence between Dipodoidea and Muroidea has limited impact on the estimated times for all nodes. The estimated date of 44 Ma (BCI 33.9–54.9) for Dipodoidea-Muroidea divergence is close to 45.4 Ma (BCI 43–49.3) when this fossil constraint was employed (Table 2). When constraints on tip rodent nodes of calibration point E, F and G were relaxed, the estimates for most nodes increased remarkably, pushing the basal divergence of Glires into the Cretaceous at 68.4 Ma (BCI 55.6–82.6) (Table 2). When only using constraint E, the most commonly used rodent calibration for mouse-rat divergence, and the two non-rodent constraints A and B, as expected [12], estimates for all nodes in Euarchontoglires incleased dramatically: 84.3 Ma (BCI 57.6–116.5) for the base of Boereoeutheria, 79 Ma (BCI 53.2–108.6) for the base of Euarchontoglires, 66.4 Ma (BCI 41–95.7) for the base of Primates, 74.7 Ma (BCI 50.6–103) for the base of Glires, and 69.7 Ma (BCI 46.5–96.1) for the base of Rodentia (Table 2). In addition, we test an alternative constraining of a minimum age of 124.6 Ma and a maximum age of 138.4 Ma for the split between placentals and marsupials to assess the sensitivity of descendant nodes to this date. The minimum age assignments were based on the Early Cretaceous *Eomaia* [40], which was previously regarded as the oldest known eutherian before the recent discovery of *Juramaia sinensis* [27] from the Late Jurassic. The maximum age was based on the basal therian *Vincelestes* [41]. The analysis shows that the change of this age resulted in slightly younger estimates of divergence times for deep nodes, but had little impact on recent nodes (Table 2). One major uncertainty in the rodent phylogeny is the position of the root of Rodentia. Based on dental morphology, Marivaux et al. [42] places Hystricomorpha as the basalmost clade of rodents. However, molecular phylogenetic studies support either Sciuromorpha, Hystricomorpha or the clade formed by Sciuromorpha and Hystricomorpha as the most basal clade of rodents, but all of these placements received relatively weak statistical support [11], [15], [43], [44]. To test whether acceptance of the alternative topologies would strongly affect our dating estimates, we conducted two alternative BEAST analyses by changing the root of Rodentia from Hystricomorpha to (1) Sciuromorpha and (2) the clade formed by Sciuromorpha and Hystricomorpha. The results showed that neither permutation had an important impact on the results, so our major conclusions remain unchanged (Figs. S4, S5).

**Table 2:** Summary of results of sensitivity test of fossil age constraints.

Nodes	Description of Nodes	Bayesian (unit: Ma)				Likelihood (unit: Ma)	
		124.6 root	No cons. D	No cons. E, F, G	No cons. C, D, F, G	180 root	No cons. E, F, G
1	Marsupialia-Placentalia	127.8 (124.6–134)	165.1 (160–175.1)	165.6 (160–176.8)	165.9 (160–177.6)	–	–
2	Base of Boereoeutheria	68.1 (58–79.3)	68.1 (54.6–83.3)	77.4 (62.2–94.9)	84.3 (57.6–116.5)	72.9	69.71
3	Perissodactyla-Carnivora	55.5 (43.6–67.7)	55.9 (43–69.7)	61.1 (45.4–78.6)	64.2 (44.2–88.3)	63.11	60.70
4	Caniformia-Feliformia	41.4 (38–47.9)	41.4 (38–47.8)	41.9 (38–49.3)	42.4 (38–50.8)	44.07	42.55
5	Primates-Glires	63.8 (55.2–73.7)	63.6 (51.4–78.1)	72.4 (58.3–88)	79 (53.2–108.6)	68.03	65.35
6	Hominoidea-Lemuroidea	54.2 (39.5–68.1)	54.6 (37.8–71.1)	62.1 (43.5–81.3)	66.4 (41–95.7)	60.08	57.86
7	Lagomorpha-Rodentia	60.5 (52.5–69.4)	60.2 (48.7–73.4)	68.4 (55.6–82.6)	74.7 (50.6–103)	64.81	62.37
8	Ochotonidae-Leporidae	34 (18.5–50.2)	34.2 (17.3–50)	38.9 (20.3–57.5)	41.5 (19.7–64.6)	39.43	38.11
9	Base of Rodentia	56.7 (49.8–64.5)	56.2 (45.3–68.1)	63.7 (52–76.5)	69.7 (46.5–96.1)	61.06	58.89
10	Base of Hystricomorpha	49.6 (42.1–57.7)	49.1 (39.7–59.5)	54.8 (44–66.8)	58.9 (39.1–83.9)	54.00	52.32
11	Hystricidae-Caviomorpha	36.87 (31.5–42.4)	36.6 (31.1–42.8)	38.7 (32–46.2)	38.8 (22.3–56.3)	35.52	35.19
12	*Octodontomys-Erethizon*	29.8 (28.5–32.2)	29.8 (28.5–32.3)	30 (28.5–32.9)	28.5 (15.9–43)	28.50	28.50
13	*Cavia-Erethizon*	24.7 (17.9–30.1)	24.8 (18.2–30.3)	24.9 (18.6–30.1)	23.5 (11.8–36.5)	25.48	25.52
14	Myodonta-Sciuromorpha	54.7 (48.3–62)	54.1 (43.4–65.7)	61.3 (50.4–73.8)	67.3 (45.6–91.9)	59.06	56.98
15	Base of Sciuromorpha	25.3 (12.8–38.6)	25.7 (12.8–39.9)	29.8 (15.8–45.6)	32.3 (14.4–53.5)	37.12	35.87
16	*Glaucomys-Tamias*	17.8 (6.7–30.6)	18.2 (7.1–31.4)	21.3 (9.2–35.6)	23 (8.2–40.9)	28.48	27.54
17	Myodonta-Castorimorpha	52.2 (46.2–58.7)	51.5 (41.1–63.1)	58.4 (47.9–70)	64.3 (44–88.6)	56.55	54.57
18	Base of Castorimorpha	45.2 (34.8–54.5)	44.6 (32.1–56.9)	50.7 (37.9–63.6)	55.4 (35.8–78.1)	51.75	49.96
19	Heteromyidae-Geomyidae	20.3 (9.7–30.8)	20 (9.2–31.6)	23.6 (11.9–36.4)	25.3 (10.8–40.7)	23.11	22.31
20	Muroidea-Dipodoidea	45 (43–48.5)	44 (33.9–54.9)	49.2 (43–57.6)	55.8 (37.3–77.1)	47.73	46.08
21	Cricetidae-Muridae	23.1 (14.1–31.9)	23.2 (14.6–33)	29.3 (19.4–39.6)	28.4 (15.3–43.5)	21.67	20.93
22	Mouse-Rat	9.5 (7.3–13)	9.5 (7.3–12.9)	16.1 (7.8–24.5)	10.3 (7.3–14.4)	11.35	10.96
24	Base of Dipodoidea	32.2 (24.8–39.3)	31.6 (22.9–40.8)	37.9 (29.6–46.8)	42.7 (27.9–60.7)	29.88	28.85
25	Zapodidae-Dipodidae	25.1 (18.7–31.8)	24.6 (17.9–31.9)	31.4 (23.5–39.2)	35.2 (22.1–50.3)	23.27	22.47
26	*Zapus-Napaeozapus*	5.6 (1.9–9.9)	5.5 (2–9.9)	6.7 (2.4–11.8)	7.4 (2.1–14)	3.63	3.50
27	Base of Dipodidae	18 (13.8–22.4)	17.9 (13.6–22.7)	25 (18.4–32.1)	28 (17.2–40.1)	16.72	16.15
28	*Salpingotus-Cardiocranius*	10.2 (9–12.3)	10.2 (9–12.3)	15.8 (8.2–23.9)	17.5 (7.9–28.4)	11.67	11.27
29	Euchoreutinae-Allactaginae	13.9 (11.2–16.8)	13.9 (11.1–17.1)	20.7 (14.9–27.1)	23.1 (14.3–33.3)	12.67	12.24
30	Dipodinae-Allactaginae	12.2 (10.5–14.5)	12.3 (10.5–14.8)	19.1 (13.7–25.2)	21.2 (13–30.8)	11.69	11.29
31	Base of Allactaginae	7.5 (5.4–9.8)	7.6 (5.3–10)	10.3 (6.7–14.3)	11.4 (6.6–17.3)	4.69	4.53
37	Base of Dipodinae	7.5 (5.2–9.8)	7.4 (5.1–9.8)	10.4 (6.4–14.5)	11.5 (6.1–17.5)	4.40	4.25

Values in parentheses are the 95% Bayesian credibility intervals. - indicates that the corresponding node was not present in the corresponding analyses. Abbreviation: cons., constraint.

The above analyses demonstrate that changes of the age of non-rodent constraints have limited impact on the estimated divergence times of nodes in the rodent tree. By contrast, removal of all three recent rodent constraints results in dramatic increase of ages for other nodes, reverting to the older divergence times estimated by earlier studies. Moreover, these results indicate that the use of a single mouse-rat constraint for the divergence time estimates for rodents can result in overestimates for all nodes in Glires.

### Likelihood

When constaints on tip rodent nodes E, F and G were relaxed, the estimated dates for all nodes change little (Table 2). This is not surprising, since age constraints on the root and deep nodes have a much bigger influence on the divergence estimates of other nodes in the program r8s [32]. Setting the age of the root to 180 Ma only caused a slight increase of the date estimates for nodes, including 64.8 Ma for the basal split of Glires, and 61.1 Ma for the basal diversification of Rodentia (Table 2). These analyses show that the influence of the root's age on the estimated dates of major rodent lineages is limited.

## Discussion

Three hypotheses have been proposed to characterize the evolutionary radiation of placental mammals: the Explosive Model puts the origin of placental orders and their intraordinal diversification shortly after the K/Pg boundary, whereas the Short Fuse Model places the origin of placental orders and intraordinal diversification in the Cretaceous, and the Long Fuse Model posits Cretaceous origins of placental orders but intraordinal diversification after the K/Pg boundary. Paleontological evidence favors the Explosive Model, suggesting that the origin and diversification of placental mammals occurred following the K/Pg extinction event that wiped out the non-avian dinosaurs and opened up many ecological niches. By contrast, recent molecular studies support either the Short Fuse or the Long Fuse Models, which suggests that continental breakup in the Late Cretaceous contributed to the origin and/or diversification of placental mammals, rather than the opening of ecological niches by differential extinction among groups. For rodents, most previous molecular studies consistently support a Short Fuse Model for them, making rodents one of the oldest placental orders which originated and diversified in the Cretaceous [11], [12], [13], [14], [15]. Because rodents lack a Cretaceous fossil record, however, there is no evidence to indicate whether their postulated diversification in the Cretaceous would have been driven by tectonic events or by other factors.

Recent phylogenetic studies, based on extensive sampling of fossil mammals, have placed all Cretaceous eutherians outside the placental crown groups [9], [33], [45]. Our estimated divergence times are consistent with a rapid radiation of major rodent lineages during the Paleocene. Therefore, our results agree with those of a few other recent molecular studies [3], [4] in supporting the hypothesis that the origin and intraordinal diversification of rodents occurred after the K/Pg Boundary about 65 Ma, following the extinction of non-avian dinosaurs. These diversification patterns are consistent with the Explosive Model as applied to mammalian orders generally, rather than with the Short Fuse Models for radiation within the orders of Rodentia.

**Figure 3:**
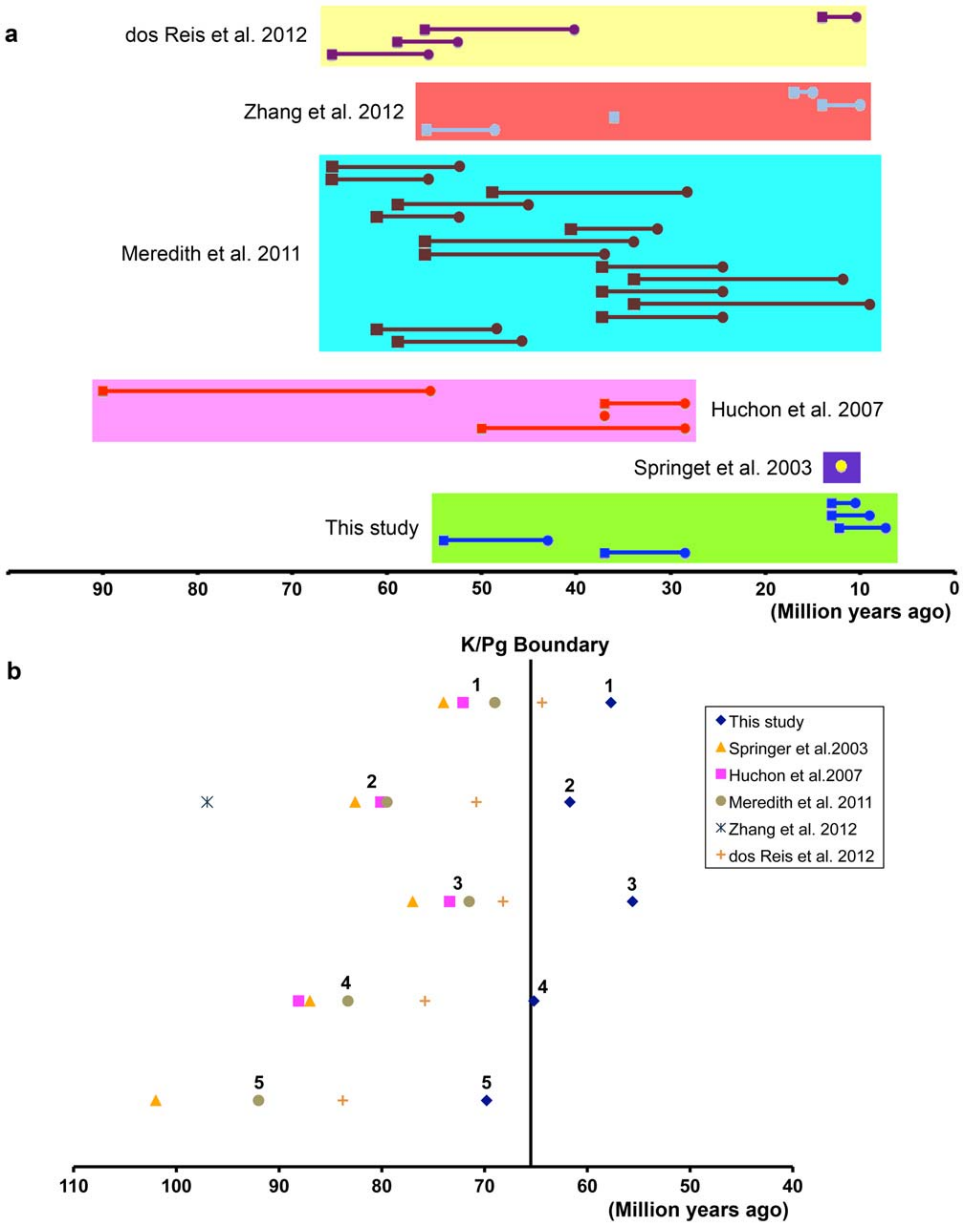
Comparisons among rodent fossil constraints used and divergence times estimated. **A**. The temporal distribution of rodent fossil constraints used. The circles and squares represent the minimum and maximum age constraints for each of the fossil calibration points, respectively. The bar connecting the circle and square shows the range between the minimum and maximum age constraints. Note that most calibration points used by Huchon et al. 2007 and Meredith et al. 2011 have much larger gap between the minimum and maximum age constraints, compared to that used by this study. **B**. Divergence time estimates obtained for major boreoeutherian lineages. The estimates of Springer et al. 2003, Huchon et al. 2007 and Meredith et al. 2011 are close to each other, and predate the K/Pg boundary. However, the divergence estimates obtained by the present study are much younger and very close to the K/Pg boundary. Numbers from 1 to 5 indicate: **1**. Base of Rodentia; **2**. Base of Glires (rodents and lagomorphs); **3**. Base of Primates; **4**. Base of Euarchontoglires; **5**. Base of Boreoeutheria.

Our study is methodologically similar to others that employed the relaxed molecular clock and multiple fossil calibration points [6], [11], [12], [14], [15], [20] (Fig. 3a). However, the divergence times we obtained, particularly those for rodents, are significantly younger than those in some recent studies that considerably predate the K/Pg boundary (Fig. 3b). Compared to the results of previous studies, our younger estimates are probably attributable to the fact that we employed multiple, internal rodent fossil constraints, which are well documented stratigraphically in a continuous sequence dated with convincing paleomagnetic chrons (see Figs. S2, S3). The study of Springer et al. (2003) [12] applied one rodent constraint, the split of mouse and rat, with a minimum age of 12 Ma (Fig. 3b). But recent increased resolution of the fossil record has decreased the minimum age constraint for mouse-rat to be around 7.3 Ma [23]. Additionally, our sensitivity test shows that the use of a single rodent calibration point can result in overestimates for all nodes in rodents. Several recent studies employed multiple rodent fossil constraints [6], [11], [15], [20]. However, their estimated divergence times for rodents are still similar to that of Springer et al. (2003), supporting the Short Fuse Model of rodent diversification (Fig. 3b).

The major difference between the fossil constraints used in this study and those used by Huchon et al. (2007) [11] and Meredith et al. (2011) [15] is that the minimum and maximum age constraints for many rodent calibration points used in both the latter studies are farther apart than the constraints for points used in our study (Fig. 3a), because of uncertainty in the paleontological and stratigraphic data associated with the fossils in question. For example, the youngest rodent fossil constraint used by Meredith et al. (2011), the split between Ctenomyidae and Ocodontidae, has a minimum age of 9.07 Ma and a maximum age of 34 Ma, for a range of 24.93 Ma. Our fossil calibration points are well constrained, with smaller amounts of time between the minimum and maximum ages (Fig. 3a). By contrast, the Zhang et al. (2012) [20] study was similar to ours in that dipodoids were densely sampled, but differed from ours in that sampling of major rodent lineages outside Dipodoidea was very limited. This lack of extensive sampling of rodent lineages may account for the fact that Zhang et al. (2012) recovered a comparatively early divergence time for the rodent-lagomorph node, consistent with earlier studies rather than with our results. Conversely, dos Reis et al. (2012) [6] carried out a study with extensive genomic sampling and broad taxonomic sampling across Placentalia, but sampled relatively few rodents of any kind. dos Reis et al. (2012) estimated the rodent-lagomorph divergence to have occurred at 70.8 Ma (Confidence Interval: 69.9–71.8), considerably earlier than the estimate recovered by our study, and the discrepancy may arise from either the lack of dense sampling within Rodentia by dos Reis et al. (2012) or the lack of extensive genomic sampling and/or broad sampling outside Rodentia in our analysis. The strengths and weaknesses of these different sampling approaches remain to be fully explored.

Our results show that reconciliation between estimates of divergence times based on molecular clock and paleontological data is possible with standard tools and genetic markers, at least for the Rodentia, the most speciose of all mammalian orders. Achieving this consistency requires a reasonable number of reliable fossil calibration points supported by a well-constrained paleontological and stratigraphic record. The consistency between our results and the paleontological record suggests that similar controversies regarding the origin and diversification of other major biological groups, including the post-K/Pg diversification of various orders of modern mammals and birds [2] and the “Cambrian Explosion” of animal phyla [46] are potentially resolvable given adequate and reliable fossil calibration points.

## Materials and Methods

### Taxon and genomic sampling

This study includes 33 rodent species across major rodent lineages and eight outgroup taxa. The taxa examined and their classification are provided in Table S2. Portions of nine unlinked the nuclear genes were sampled, including alpha 2B adrenergic receptor (A2AB), cannabinoid receptor 1 (CNR1), growth hormone receptor (GHR), interphotoreceptor retinoid binding protein (IRBP), breast cancer susceptibility (BRCA1), von Willebrand factor (vWF), ATPase, Cu++ transporting, alpha polypeptide (ATP7A), 3′-UTR region of cAMP responsive element modulator (Crem), recombination activating gene 2 (RAG2). Detailed information of these loci is provided in Table S3.

Genomic DNA was prepared from either muscle or liver tissue samples using DNeasy Tissue Kit (Qiagen, inc). PCR reactions were undertaken in 25-µL volumes with the following conditions: 94°C (5–10 min); 35 cycles of 94°C (45 s); 55°C (45 s); 72°C (40–60 s); 72°C (5–10 min). Sequence data were collected on ABI 3730 DNA Analyzers for both directions subsequent to Big Dye chemistry. The primers for both PCR and sequencing reactions are identified in Table S4.

DNA sequences were aligned using Kalign as implemented in the program eBioX (http://www.ebioinformatics.org) under default conditions, and refined manually using MacClade 4.06 [47]. Ambiguous sites including potentially heterozygous sites were encoded based on IUPAC Ambiguity protocol. The GenBank accession numbers for each of the sequence data are provided in Table S5.

### Phylogenetic analysis

Phylogenetic trees were constructed using maximum likelihood and Bayesian criteria. The Akaike information criterion was used to determine the best substitution models of sequence evolution based on the results from MODELTEST 3.07 [48] (Table S3). The maximum likelihood estimates for the best tree were performed with the program PhyML 3.0 [31] for the concatenated dataset with 100 bootstrap replicates.

Bayesian inference of phylogeny was performed with the program MrBayes 3.1.2 [49]. We performed four independent runs under identical conditions with partitions defined for each of the nine loci evolving with independent model parameters. For each analysis, one run was performed with four chains, and was sampled every 2000 generations for fifty million generations after a burn-in cycle of 5000 trees. The convergence of each run was examined with the program Tracer 1.5 [29].

### Test of molecular rate heterogeneity

The levels of molecular rate heterogeneity for the concatenated dataset and for each of the loci were examined in the program BEAST 1.5.4 [29]. When running under the uncorrelated relaxed lognormal clock model, BEAST can measure the ucld.stdev parameter, which can determine how clock-like the DNA dataset is. The dataset is strictly clock-like if the ucld.stdev parameter is 0, and the dataset has substantial rate heterogeneity among lineages if the parameter is greater than 1. The dataset has a moderate level of heterogeneity if the ucld.stdev parameter lies between 0 and 1 [29]. For each individual locus, the test incorporated in BEAST was performed using five million generations sampled every 500 generations. For the concatenated dataset, BEAST run was performed using 40 million generations, sampled every 500 generations for two independent runs.

### Molecular estimates of divergence times

We employed two approaches to estimates divergence times of each node: a Bayesian method as implemented in the program BEAST 1.5.4, and a penalized likelihood method in the program r8s 1.71 [32].

Bayesian analyses of molecular dating were estimated for the combined dataset with the substitution models for each gene partition. The relaxed molecular clock model was chosen for all BEAST analyses [50], since the estimated value of ucld.parameter is 0.57 for the concatenated DNA dataset. Each run of BEAST analyses comprised forty million generations, sampled either every 500 generations or 1000 generations for two independent runs. The output files of the two independent analyses were combined using LogCombiner 1.5.4 [29] to produce the final results. Each run was examined with the program Tracer 1.5 [29] for convergence.

The program r8s [32] was used to compare maximum likelihood results with those obtained from BEAST. The r8s analyses were performed using the best maximum likelihood tree calculated in PhyML. We used the penalized likelihood model, the log penalty function and the truncated Newton algorithm based on the recommendation of the developer [32]. The optimal smoothing parameter was determined by a cross-validation run with r8s. For the nodes used as fossil age constraints, the check-gradient function was conducted to determine if the estimated values of divergence timings of these nodes fall beyond the age constraints. The date of root (marsupial-placental) was fixed at 160 and 180 Ma in order to assess the sensitivity of descendant nodes to the age of the root. Since the use of the two different root ages do not have significant impact on the estimated ages of other nodes (Table 2), this study used 160 as the age of the root for all r8s analyses.

### Fossil constraints

We applied seven fossil age constraints for the molecular dating analyses in this study. Minimum age constraints were based on the earliest known fossil record of a member of one of the divergent lineages. Where possible, the maximum age constraints are based on the age of the youngest well-sampled horizon that does not contain any members of the divergent lineages, in a stratigraphic sequence in which members of these lineages subsequently appear. When a stratigraphic sequence suitable for setting a particular upper bound by this method was not available, the age of the oldest member of the stem lineage leading up to the divergence was used as the upper bound. For the program BEAST, these calibration points were set as soft constraints with upper and lower bounds that allow for a 2.5% chance of lying beyond each user-input bound. The r8s program only allows the fossil calibrations to be set as a hard bound. Fossil constraints are as follows (Fig. 2):

- we assigned a minimum age of 160 Ma and a maximum age of 190 Ma for the divergence between marsupials and placentals, based on the earliest known placental mammal *Juramaia sinensis* [27] and the basal mammal *Hadrocodium* [26].
- We assigned a minimum age of 38 Ma and a maximum age of 61.7 Ma for the divergence between caniforms and feliforms, based on the oldest known crown carnivoran *Hesperocyon* and *Daphoenus* from the Late Eocene [28] and the oldest stem carnivore *Protictis schaffi* from the early Paleocene [28], respectively.
- We assigned a minimum age of 28.5 Ma and a maximum age of 37 Ma for the divergence between *Octodontomys* and the clade formed by *Cavia* and *Erethizon*, based on the oldest fossil record of Caviomorpha from the Late Eocene [25] and the oldest fossil erethizontid (*Steiromys* sp.) from the mid-Oligocene [24].
- The earliest muroid is *Pappocricetodon* [22], and the earliest dipodoid is *Primisminthus* and *Banyuesminthus* [21]. All the above species emerge in the middle part of the Eocene of China. *Erlianomys*, which is from the lower part of the Arshanto Formation in Nuhetingboerhe of Inner Mongolia, China, represents the earliest fossil record of myodonts [19]. Based on recent magneto-stratigraphic analyses, the Nuhetingboerhe section was dated to the early part of the Early Eocene [51] (Fig. S3). Consistent with these fossil and stratigraphic results, we assigned a minimum age of 43 Ma and a maximum age of 54 Ma for the divergence between muroids and dipodoids.
- We assigned a minimum age of 7.3 Ma and a maximum age of 12.2 Ma for the divergence between mice and rats, based on the occurence of the earliest known mouse *Mus* sp. and the earliest known *Prognomys* from the late Miocene of Pakistan [23].
- We assigned a minimum age of 9 Ma for the divergence between the two cardiocraniine genera *Cardiocranius* and *Salpingotus*, based on the occurence of the earliest known *Cardiocranius* (*C. pussillus*) and the earliest known *Salpingotus* (*S. primitivus*) from the Late Miocene of China [18].
- We assigned a minimum age of 10.5 Ma for the divergence between the two dipodoid subfamilies Dipodinae and Allactaginae, based on the occurence of the earliest known dental fossils of Dipodinae from the middle bed of the Dingshanyanchi Formation, Xinjiang, China (Fig. S1). The cheek teeth of the Dingshanyanchi species lack the mesoloph and mesocone on the upper molars, and have no mesolophid and mesoconid on the lower molars. Cusps on the labial and lingual sides of each molar show an alternating, rather than opposite, arrangement. The anteroloph of M2 and ectolophid of the lower molars are oriented oblique to the longitudinal axis. These dental features represent synapomorphies of dipodine molars. According to stratigraphic and palaeomagnetic results, the middle bed of the Dingshanyanchi Formation falls toward the base of the long normal magnetic chron C5n.2n, and thus dates to the earliest part of the Late Miocene [52] (Fig. S2).

We set a maximum age of 13 Ma for the divergence between Dipodinae and Allactaginae and the divergence between *Salpingotus* and *Cardiocranius*. The Middle Miocene deposits are well exposed and have been extensively sampled in northern China and surrounding areas [53], [54], [55], [56]. The only Dipodidae that can be found during the Middle Miocene is *Protalactaga*, a primitive genus. These deposits produce no species of extant dipodid subfamilies – Dipodinae, Allactaginae, Euchoreutinae and Cardiocraniinae – not even in the northern Junggar Basin of Xinjiang, which has a continuous geological sequence ranging from the Late Oligocene to the Late Miocene and where screen washing has been applied for decades [52], [53], [55]. On this basis, the maximal boundary for these two divergence events was placed in the middle part of the Middle Miocene.

## Supporting Information

Figure S1 **Occlusal view of molars of the earliest fossil representative of Dipodinae from the Dingshanyanchi Formation.** A. right m1 (IVPP V 16905.2). B. right m2 (IVPP V 16905.3). C. right M2 (IVPP V 16905.1). D. left m3 (IVPP V 16905.5). E. right m3 (IVPP V 16905.4).
(TIF)

Figure S2 **Magneto-stratigaphic sequence of the Dingshanyanchi Formation, Xinjiang, China.** This figure was based on and modified from Sun et al. 2010 [52]. Blue arrow indicates the layer that produced the earliest fossil representative of Dipodinae showing in Figure S1.
(TIF)

Figure S3 **Magneto-stratigraphic sequence of the Arshanto Formation.** This figure was based on and modified from Sun et al. 2009 [51]. Blue arrow indicates the layer that produced the earliest known myodont fossil, *Erlianomys*.
(TIF)

Figure S4 **Divergence times obtained from Bayesian estimates based on the alternative topology with Sciuromorpha as the basal clade of Rodentia.** Note that the estimated divergence times support a post-Cretaceous origin and diversification of Rodentia.
(TIF)

Figure S5 **Divergence times obtained from Bayesian estimates based on the alternative topology with the clade of Sciuromorpha and Hystricomorpha as the basal clade of Rodentia.** Note that the estimated divergence times support a post-Cretaceous origin and diversification of Rodentia.
(TIF)

Table S1 Results of the test of molecular rate heterogeneity. The ucld.stdev parameters for each locus and the concatenated, partitioned data-set estimated by BEAST. Abbreviations: 95% C. I. = 95% Confidence Interval; ESS = Effective Sample Size.
(PDF)

Table S2 List of taxon sampling for this study. Abbreviations: MVZ = Museum of Vertebrate Zoology, University of California, Berkeley; MCZ = Museum of Comparative Zoology, Harvard University; AMNH = American Museum of Natural History; NMNH = National Museum of Natural History, Smithsonian Institution.
(PDF)

Table S3 Characteristics of genes included show the AIC weights supporting the best model for each entry.
(PDF)

Table S4 Primer.
(PDF)

Table S5 List of GenBank accession numbers.
(PDF)

Table S6 Comparisons of divergence times for major nodes estimated using BEAST with full taxa and with a tree that is free of NDE by reducing taxon sampling in the subfamilies Dipodinae and Allactaginae. Values in parentheses are the 95% Bayesian credibility intervals. Note that these two analyses produced similar estimates of divergence times for major nodes. Statistical test shows that there is no significant difference between these two time estimates (t-test, p-value = 0.954).
(PDF)
