# Supplementary figures and images for: Molecular and Paleontological Evidence for a Post-Cretaceous Origin of Rodents
Source: PLoS One. 2012 Oct 5;7(10):e46445. doi: 10.1371/journal.pone.0046445 (PMC3465340; doi:10.1371/journal.pone.0046445)

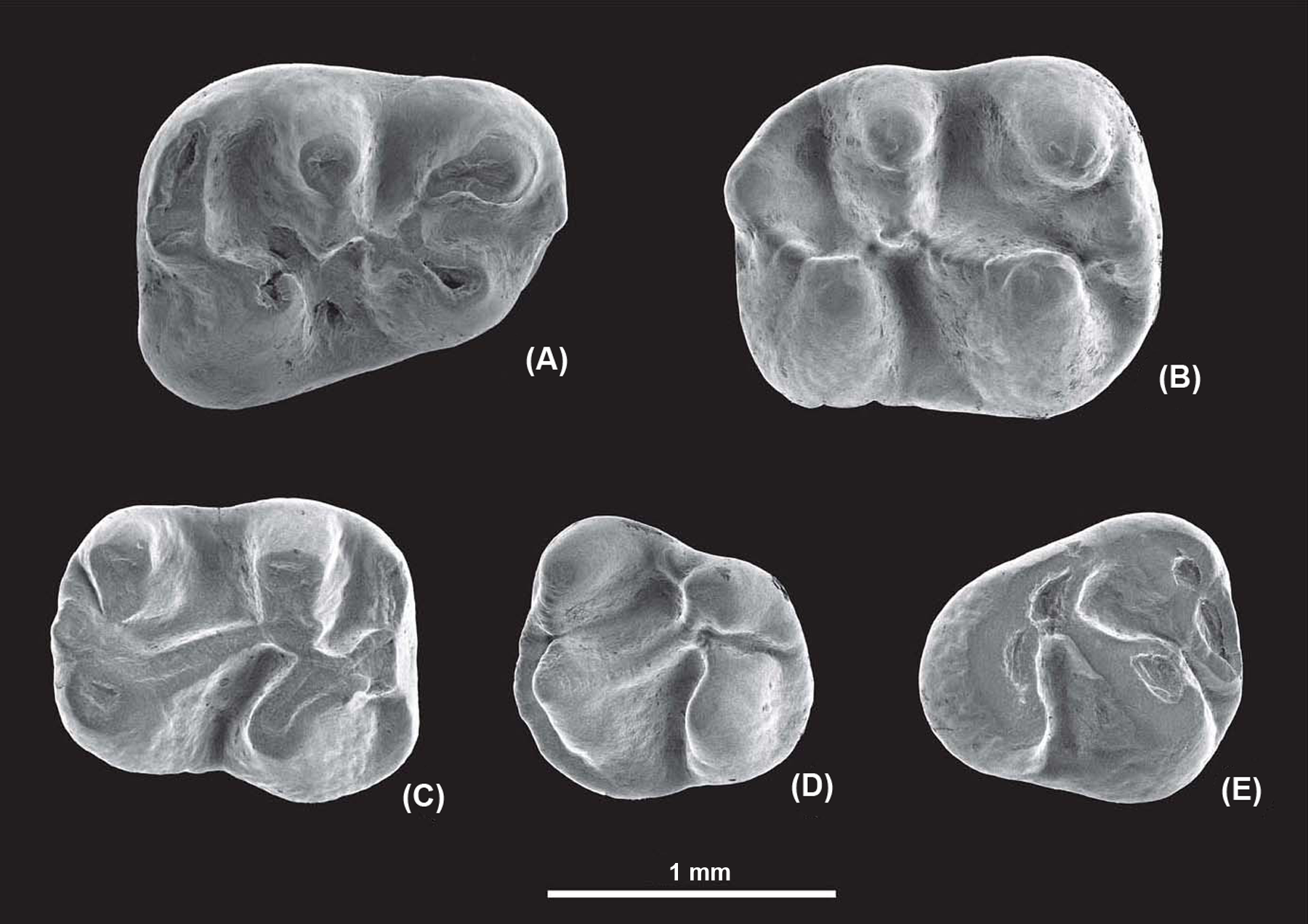

Supplement: Figure S1 — Occlusal view of molars of the earliest fossil representative of Dipodinae from the Dingshanyanchi Formation. A. right m1 (IVPP V 16905.2). B. right m2 (IVPP V 16905.3). C. right M2 (IVPP V 16905.1). D. left m3 (IVPP V 16905.5). E. right m3 (IVPP V 16905.4). (TIF) [file pone.0046445.s001.tif]

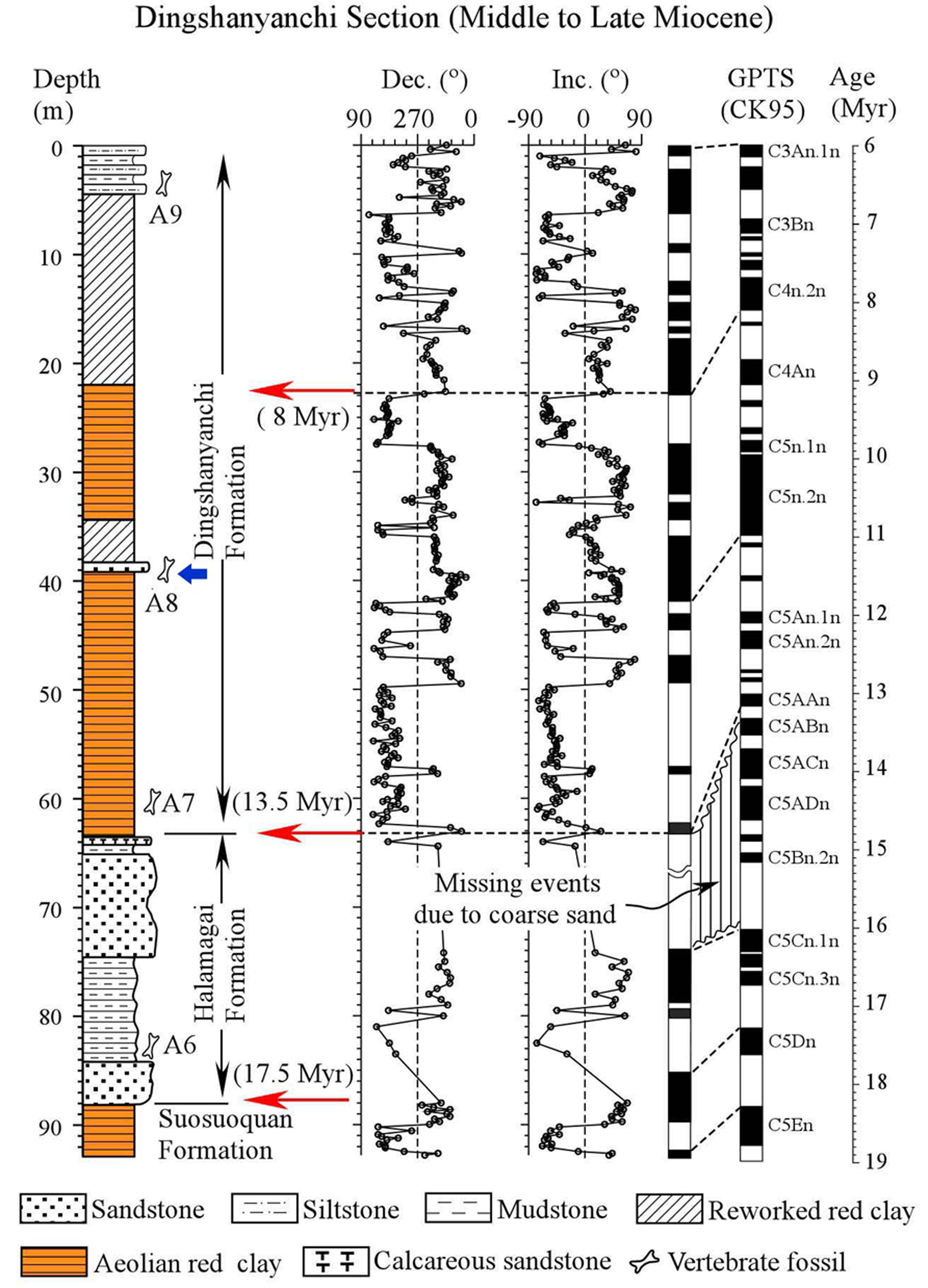

Supplement: Figure S2 — Magneto-stratigaphic sequence of the Dingshanyanchi Formation, Xinjiang, China. This figure was based on and modified from Sun et al. 2010 [52]. Blue arrow indicates the layer that produced the earliest fossil representative of Dipodinae showing in Figure S1. (TIF) [file pone.0046445.s002.tif]

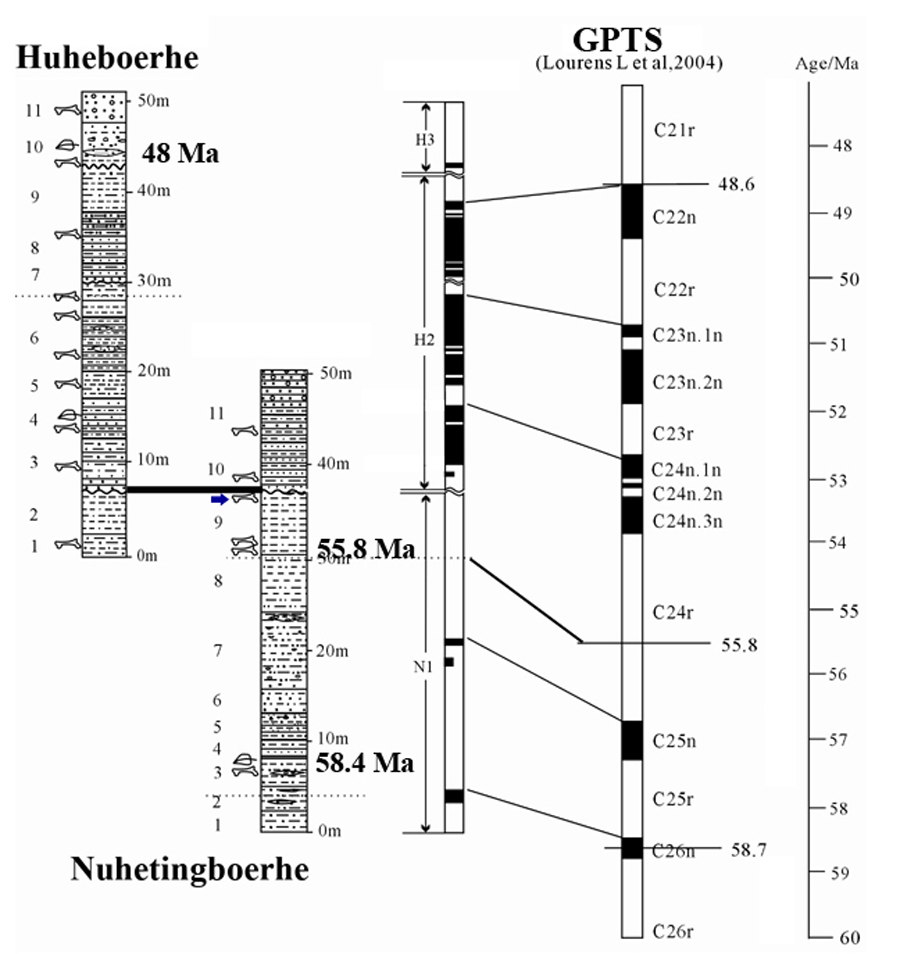

Supplement: Figure S3 — Magneto-stratigraphic sequence of the Arshanto Formation. This figure was based on and modified from Sun et al. 2009 [51]. Blue arrow indicates the layer that produced the earliest known myodont fossil, Erlianomys. (TIF) [file pone.0046445.s003.tif]

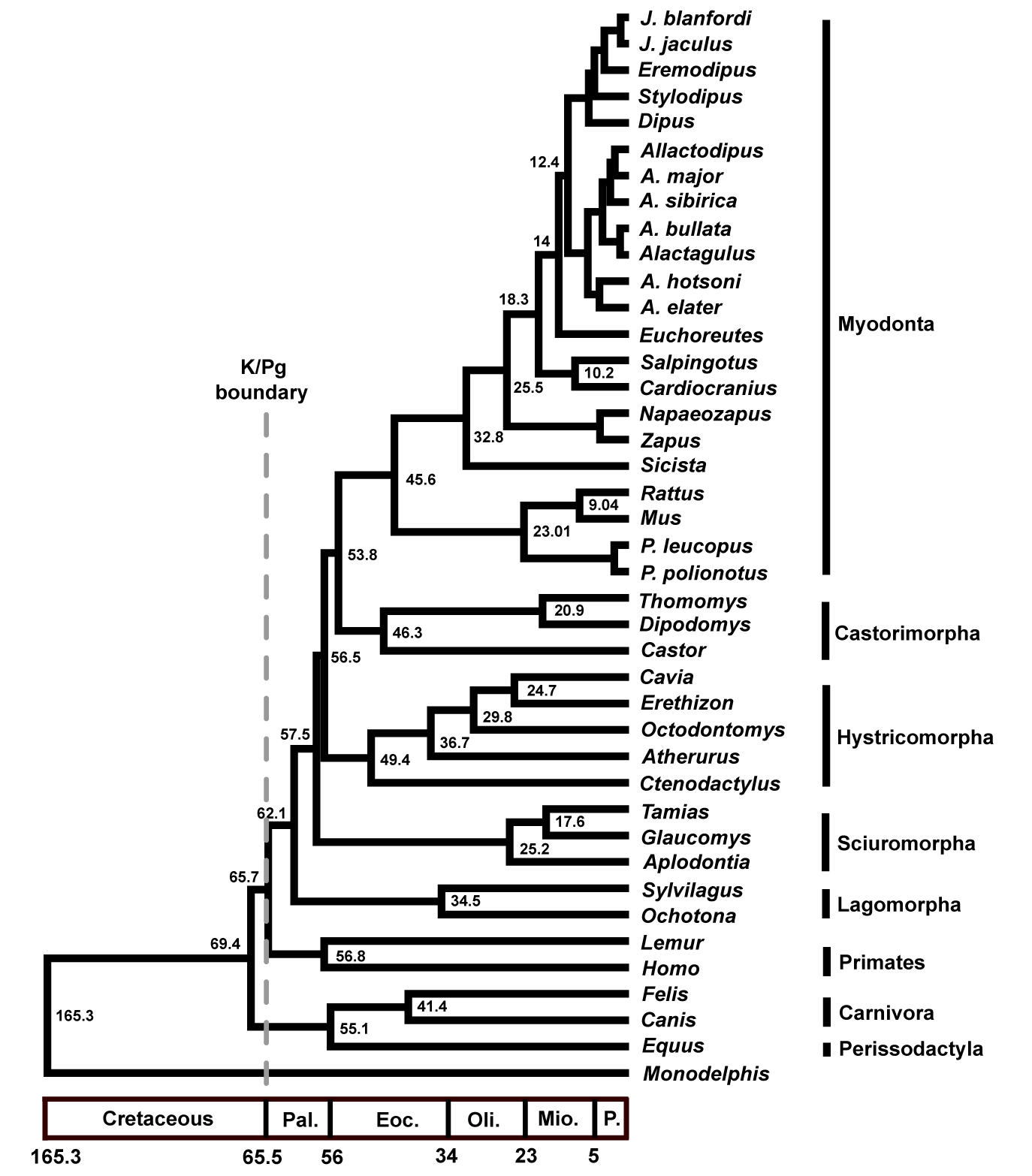

Supplement: Figure S4 — Divergence times obtained from Bayesian estimates based on the alternative topology with Sciuromorpha as the basal clade of Rodentia. Note that the estimated divergence times support a post-Cretaceous origin and diversification of Rodentia. (TIF) [file pone.0046445.s004.tif]

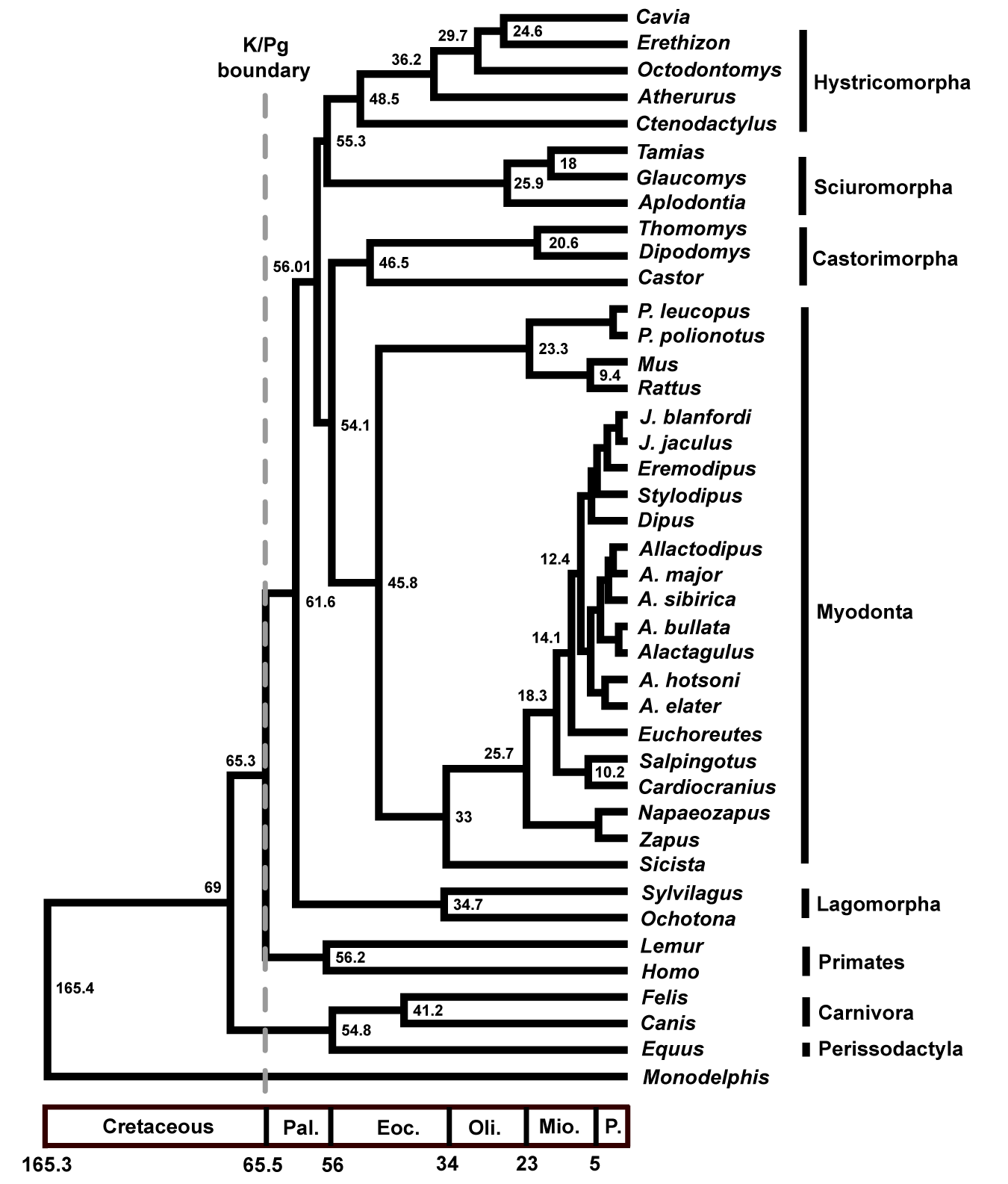

Supplement: Figure S5 — Divergence times obtained from Bayesian estimates based on the alternative topology with the clade of Sciuromorpha and Hystricomorpha as the basal clade of Rodentia. Note that the estimated divergence times support a post-Cretaceous origin and diversification of Rodentia. (TIF) [file pone.0046445.s005.tif]
