# Supplementary material for: Molecular and Paleontological Evidence for a Post-Cretaceous Origin of Rodents
Source: PLoS One. 2012 Oct 5;7(10):e46445. doi: 10.1371/journal.pone.0046445 (PMC3465340; doi:10.1371/journal.pone.0046445)
Supplement: Table S1 — Results of the test of molecular rate heterogeneity. The ucld.stdev parameters for each locus and the concatenated, partitioned data-set estimated by BEAST. Abbreviations: 95% C. I. = 95% Confidence Interval; ESS = Effective Sample Size. (PDF) [file pone.0046445.s006.pdf]

Table S1. Results of the test of molecular rate heterogeneity. The ucld.stdev parameters for each locus and the concatenated, partitioned data-set estimated by BEAST. Abbreviations: 95% C. I. = 95% Confidence Interval; ESS = Effective Sample Size.

Locus	ucld.stdev	95% C. I.	ESS
A2AB	0.772	0.53-1.02	353
CNR1	1.788	1.35-2.19	100
GHR	0.649	0.47-0.83	85
IRBP	0.481	0.34-0.64	485
BRCA1	0.781	0.59-0.98	190
vWF	0.628	0.46-0.8	362
ATP7A	0.63	0.45-0.82	201
Crem 3'UTR	0.807	0.56-1.09	208
RAG2	0.704	0.51-0.93	315
Concatenated	0.57	0.45-0.71	383
