# Supplementary material for: Molecular and Paleontological Evidence for a Post-Cretaceous Origin of Rodents
Source: PLoS One. 2012 Oct 5;7(10):e46445. doi: 10.1371/journal.pone.0046445 (PMC3465340; doi:10.1371/journal.pone.0046445)
Supplement: Table S2 — List of taxon sampling for this study. Abbreviations: MVZ = Museum of Vertebrate Zoology, University of California, Berkeley; MCZ = Museum of Comparative Zoology, Harvard University; AMNH = American Museum of Natural History; NMNH = National Museum of Natural History, Smithsonian Institution. (PDF) [file pone.0046445.s007.pdf]

Table S2. List of taxon sampling for this study. Abbreviations: MVZ = Museum of Vertebrate Zoology, University of California, Berkeley; MCZ = Museum of Comparative Zoology, Harvard University; AMNH = American Museum of Natural History; NMNH = National Museum of Natural History, Smithsonia Institution.

Classification	Genus	Species	Source
**Eutheria**			
**Rodentia**			
Dipodidae	*Jaculus*	*jaculus*	Egypt; GenBank
	Jaculus	*blanfordi*	MVZ192036
	Allactaga	*hotsoni*	MVZ1992077
	Cardiocranius	*paradoxus*	MVZ175166
	Eremodipus	*lichtensteini*	Russia
	Allactaga	*major*	Russia
	Allactodipus	*bobrinskii*	Russia
	Alactagulus	*pumilio*	Xinjiang, China
	Dipus	*sagitta*	Nei Mongol, China
	Allactaga	*sibirica*	Nei Mongol, China
	Allactaga	*bullata*	Xinjiang, China
	Stylodipus	*telum*	Xinjiang, China
	Euchoreutes	*naso*	Xinjiang, China
	Allactaga	*elater*	Xinjiang, China
	Salpingotus	*kozlovi*	Xinjiang, China
Zapodidae	*Zapus*	*hudsonius*	MCZ, Harvard
	Napaeozapus	*insignis*	MCZ, Harvard
Sicistidae	*Sicista*	*S. tianshanica,* Xinjiang, China; *S. kazbegica*, GenBank	
Muridae	*Mus*	*musculus*	GenBank
	Rattus	*norvegicus*	GenBank
Cricetidae	*Peromyscus*	*polionotus*	MCZ64944
	Peromyscus	*leucopus*	MCZ63171
Heteromyidae	*Dipodomys*	*D. ordii*, MCZ; *D. spectabilis*, *D. merriami*, *D. heermanni*, GenBank	
Castoridae	*Castor*	*Canadensis*	GenBank
Sciuridae	*Tamias*	*Striatus*	MCZ64175; GenBank
	Glaucomys	*Volans*	MCZ66196; GenBank
Octodontidae	*Octodontomys*	*gliroides*	AMCC103810, AMNH; GenBank
Hystricidae	*Atherurus*	*macrourus*	AMCC125125, AMNH; GenBank
Erethizontidae	*Erethizon*	*dorsatum*	GenBank
Geomyidae	*Thomomys*	*T. bottae,* MVZ174912; *T. talpoides*, GenBank	
Caviidae	*Cavia*	*C. aperea,* MVZ183276; *C. porcellus*, *C. tschudii*, GenBank	
Ctenodactylidae	*Ctenodactylus*	*C. gundi,* MVZ201014; *C. vali*, GenBank	
Aplodontidae	*Aplodontia*	*rufa*	MVZ198835; GenBank
**Lagomorpha**			
Ochotonidae	*Ochotona*	*O. princeps*, USNM568433, NMNH; *O. hyperborea*, GenBank	
Leporidae	*Sylvilagus*	*floridanus*	USNM567962, NMNH; GenBank
**Primates**			

Lemuridae	*Lemur*	*catta*	AMCC100346, AMNH; GenBank
Hominidae	*Homo*	*sapiens*	GenBank
**Carnivora**			
Canidae	*Canis*	*familiaris*	GenBank
Felidae	*Felis*	*catus*	GenBank
**Perissodactyla**			
Equidae	*Equus*	*cabailus*	GenBank
**Marsupialia**			
Didelphidae	*Monodelphis*	*domestica*	GenBank
