# Supplementary material for: Molecular and Paleontological Evidence for a Post-Cretaceous Origin of Rodents
Source: PLoS One. 2012 Oct 5;7(10):e46445. doi: 10.1371/journal.pone.0046445 (PMC3465340; doi:10.1371/journal.pone.0046445)
Supplement: Table S3 — Characteristics of genes included show the AIC weights supporting the best model for each entry. (PDF) [file pone.0046445.s008.pdf]

Table S3. Characteristics of genes included show the AIC weights supporting the best model for each entry.

Genes	Length (bp)	Variable sites	Parsimony informative sites	Best Model
A2AB	504	197	136	HKY+I+G
CNR1	469	258	118	GTR+1+G
GHR	650	470	346	GTR+I+G
IRBP	541	330	252	SYM+I+G
BRCA1	573	498	413	HKY+I+G
vWF	477	301	219	HKY+I+G
ATP7A	596	346	237	HKY+I+G
Crem	437	269	187	SYM+G
RAG2	438	272	193	HKY+G
Concatenated data set	4685	2941	2101	GTR+I+G
