# Supplementary material for: Molecular and Paleontological Evidence for a Post-Cretaceous Origin of Rodents
Source: PLoS One. 2012 Oct 5;7(10):e46445. doi: 10.1371/journal.pone.0046445 (PMC3465340; doi:10.1371/journal.pone.0046445)
Supplement: Table S4 — Primer. (PDF) [file pone.0046445.s009.pdf]

Table S4. Primer

Locus	Part	Primer Name	Sequence (5' - 3')	Source
A2AB	Exon 1	A2AB-11F	CATCACCTTCCTCATCCTCTTCAC	This study
		A2AB-15R	ATCAGGTAGATGCGMAGGTAGAC	
BRCA1	Exon 11	BRCA1-601F	AAGCAAACAGTCTGGCTTAGAAAGG	This study
		BRCA1-501F	TGCCARCTYATTACAGCVTGRGA	
		BRCA1-64R	CTTGATAAAATCCTCAGGTTGAAGGC	
		BRCA1-620R	YRKGGYTCARRTKRGGGMRGC	
CNR1		CNR1-11F	AGTGTGGGGAGAACTTCATGGACAT	This study
		CNR1-5R	CAGACTGMAGCTTCTTGCAGTTCC	
GHR	Exon 10	GHR-50F	TTCTAYARYGATGACTCYTGGGT	Adkins et al. 2001[9]
		GHR-750R	GTAAGGCTTTCTGTGGTGATRTAA	
		GHR-33F	TTCTACAATGATGACTCTTGGGTTGAGTT	This study
		GHR-303F	CAGCACTACCCTAACAGAGGAAGA	
		GHR-301R	GGGCATAAAAGTCAATGTTGGCCAG	
		GHR-35R	TTGAGTATGAGGCCCTGTGGAGA	
IRBP	Exon 1	IRBP-11F	CCACGCYTGGTCATCTCCTAYGA	This study
		IRBP-15R	TGGCCTATTCTCAGCTTCTGGAG	
vWF	Exon 28	vWF-101F	TGTCAACCTCACCTGTGAAGCCTG	Huchon et al. 1999[10]
		vWF-103R	GACCTTCTTCTTCTTCAAGCCCTGG	This study
		vWF-F101g	TGTSAACCTYACSTGTGAAGCCTG	
		vWF-113R	RACCTTCTTCTTCTTSARDCCYTGG	
ATP7A		ATP7A-11F	TCCCTGGACAATCAAGAAGC	Murphy et al. 2001[11]
		ATP7A-15R	AAGGTAGCATCAAATCCCATGT	
Crem	3'-UTR	Crem-21F	CTTGTAGAAGGGACCCTTGT	This study
		Crem-19R	ACATTCTTTTGCCACTTCCCT	
		Crem-23F	GTCTGCCCTCTTGCTTCTA	
		Crem-17R	TCATGCACTAAGCCAAGCTG	
RAG2		RAG2-21F	CGAGGGAAAAGTATGGGTGT	This study
		RAG2-19R	GGTATCACTTCTGGCAATGG	
		RAG2-23F	ACATACTTCCAGAACTTCAGG	
		RAG2-17R	TTCTGTGGTTCTTTGGGTAGA	
