# Supplementary material for: Molecular and Paleontological Evidence for a Post-Cretaceous Origin of Rodents
Source: PLoS One. 2012 Oct 5;7(10):e46445. doi: 10.1371/journal.pone.0046445 (PMC3465340; doi:10.1371/journal.pone.0046445)
Supplement: Table S5 — List of GenBank accession numbers. (PDF) [file pone.0046445.s010.pdf]

Table S5. List of the GenBank accession numbers

Genus	Species	Accession Number
Jaculus	*jaculus*	A2AB: JF938725; CNR1: JF938804; GHR: JF938854; IRBP: JF938880; BRCA1: JF938777; vWF: AJ297765; ATP7A: JF938751; Crem: JF938827; RAG2: JF938903
	blanfordi	A2AB: JF938726; CNR1: JF938805; GHR: JF938855; IRBP: JF938881; BRCA1: JF938778; vWF: JF938703; ATP7A: JF938752; Crem: JF938828; RAG2 Jb JF938904
Allactaga	*hotsoni*	A2AB: JF938728; CNR1: JF938807; GHR: JF938857; IRBP: JF938883; BRCA1: JF938778; vWF: JF938705; ATP7A: JF938754; Crem: JF938830; RAG2: JF938906
	elater	A2AB: JF938727; CNR1: JF938806; GHR: JF938856; IRBP: JF938882; BRCA1: JF938779; vWF: JF938704; ATP7A: JF938753; Crem: JF938829; RAG2: JF938905
	sibirica	A2AB: JF938716; CNR1: JF938795; GHR: JF938845; IRBP: JF938871; BRCA1: JF938768; vWF: JF938694; ATP7A: JF938742; Crem: JF938818; RAG2: JF938894
	major	A2AB: JF938717; CNR1: JF938796; GHR: JF938846; IRBP : JF938872; BRCA1: F938769; vWF: JF938695; ATP7A: JF938743; Crem: JF938819; RAG2: JF938895
	bullata	A2AB: JF938722; CNR1: JF938801; GHR: JF938851; IRBP: JF938877; BRCA1: JF938774; vWF: JF938700; ATP7A: JF938748; Crem: JF938824; RAG2: JF938900
Alactagulus	*pumilio*	A2AB: JF938718; CNR1: JF938797; GHR: JF938847; IRBP: JF938873; BRCA1: JF938770; vWF: JF938696; ATP7A: JF938744; Crem: JF938820; RAG2:JF938896
Allactodipus	*bobrinskii*	A2AB: JF938719; CNR1: JF938798; GHR: JF938848; IRBP: JF938874; BRCA1: JF938771; vWF: JF938697; ATP7A: JF938745; Crem: JF938821; RAG2: JF938897
Euchoreutes	*naso*	A2AB: JF938724; CNR1: JF938803; GHR: JF938853; IRBP: JF938879; BRCA1: JF938776; vWF: JF938702; ATP7A: JF938750; Crem: JF938826; RAG2:JF938902
Eremodipus	*lichtensteini*	A2AB: JF938721; CNR1: JF938800; GHR: JF938850; IRBP: JF938876; BRCA1: JF938773; vWF: JF938699; ATP7A: JF938747; Crem: JF938823; RAG2: JF938899
Dipus	*sagitta*	A2AB: JF938720; CNR1: JF938799; GHR: JF938849; IRBP: JF938875; BRCA1: JF938772; vWF: JF938698; ATP7A: JF938746; Crem: JF938822; RAG2: JF938898
Stylodipus	*telum*	A2AB: JF938723; CNR1: JF938802; GHR: JF938852; IRBP: JF938878; BRCA1: JF938775; vWF: JF938701; ATP7A: JF938749; Crem: JF938825; RAG2: JF938901
Cardiocranius	*paradoxus*	A2AB: JF938732; CNR1: JF938811; GHR: JF938862; IRBP: JF938887; BRCA1: JF938785; vWF: JF938709; ATP7A: JF938758; Crem: JF938834; RAG2: JF938911
Salpingotus	*kozlovi*	A2ABJF938729; CNR1: JF938808; GHR: JF938858; IRBP: JF938884; BRCA1: JF938781; vWF: JF938706; ATP7A: JF938755; Crem: JF938831; RAG2: JF938907
Zapus	*hudsonius*	A2AB: JF938714; CNR1: JF938793; GHR: JF938843; IRBP: JF938869; BRCA1: JF938766; vWF: JF938692; ATP7A: JF938740; Crem: JF938816; RAG2: JF938892
Napaeozapus	*insignis*	A2AB: JF938715; CNR1: JF938794; GHR: JF938844; IRBP: JF938870; BRCA1: JF938767; vWF: JF938693; ATP7A: JF938741; Crem: JF938817; RAG2: JF938893

Sicista	*tianshanica*	A2AB: JF938713; CNR1: JF938792; GHR: JF938842; IRBP: JF938868; BRCA1: JF938765; ATP7A: JF938739; Crem: JF938815; RAG2: JF938891
	kazbegica	vWF: AJ297764
Mus	*musculus*	A2AB: NM009633; CNR1: AY522554; GHR: NM010284; IRBP: AB125808; BRCA1: NM009764; vWF: AF539800; ATP7A: AY011397; Crem: M60284; RAG2: AY011940
Rattus	*norvegicus*	A2AB: NM138505; CNR1: NM012784; GHR: X16726; IRBP: AJ429134; BRCA1: NM012514; vWF: AJ224673; ATP7A: AY011398; Crem: NM001110860; RAG2: AY011941
Peromyscus	*polionotus*	A2AB: JF938730; CNR1: JF938809; GHR: JF938859; IRBP: JF938885; BRCA1: JF938782; vWF: JF938707; ATP7A: JF938756; Crem: JF938832; RAG2: JF938908
Peromyscus	*leucopus*	A2AB: JF938731; CNR1: JF938810; GHR: JF938860; IRBP: JF938886; BRCA1: JF938783; vWF: JF938708; ATP7A: JF938757; Crem: JF938833; RAG2: JF938909
Dipodomys	*ordii*	GHR: JF938861; BRCA1: JF938784; RAG2: JF938910
	merriami	A2AB: AJ427261; IRBP: AJ427233; vWF: AJ427226
	spectabilis	CNR1: AY303183
	heermanni	ATP7A: AY011402; Crem: AY011648
Castor	*canadensis*	A2AB: AJ427260; CNR1: AY011576; GHR: AF332026; IRBP: AF297279; BRCA1: AF540622; vWF: AJ427228; ATP7A: AY011394; Crem: AY011640; RAG2: AY011937
Tamias	*striatus*	A2AB: JF938733; CNR1: AY011575; GHR: JF938863; IRBP: AF297282; BRCA1: JF938786; vWF: JF938710; ATP7A: AY011393; Crem: AY011639; RAG2: AY011936.1
Glaucomys	*volans*	A2AB: JF938734; CNR1: JF938812; GHR: JF938864; IRBP: AY227598; BRCA1: AF284003; vWF: AJ224667; ATP7A: JF938759; Crem: JF938835; RAG2: JF938912
Octodontomys	*gliroides*	A2AB: JF938735; CNR1: JF938813; GHR: AF520649; IRBP: JF938888; BRCA1: JF938787; vWF: JF938711; ATP7A: JF938760; Crem: JF938836; RAG2: JF938913
Atherurus	*macrourus*	A2AB: JF938736; CNR1: JF938814; GHR: JF938865; IRBP: JF938889; BRCA1: JF938788; vWF: AJ251131; ATP7A: JF938761; Crem: JF938837; RAG2: JF938914
Erethizon	*dorsatum*	A2AB: AJ427270; CNR1: AY303186; GHR: AF332037; IRBP: AJ427249; BRCA1: AF540626; vWF: AJ251135; ATP7A: AY011401; Crem: AY011647; RAG2: AY011944
Thomomys	*bottae*	CNR1: AY303196; GHR: AM407911; IRBP: AF297277; BRCA1: JF938790; ATP7A: JF938762; Crem: JF938841; RAG2: AY303215
	talpoides	A2AB: AJ427262; vWF: AJ427227
Cavia	*aperea*	GHR: AF433930; BRCA1: JF938791
	porcellus	A2AB: AJ271336; IRBP: AJ427248; vWF: AJ224663
	tschudii	CNR1: AY011586; ATP7A: AY011404; Crem: AY011650; RAG2:AY011947
Ctenodactylus	*gundi*	A2AB: JF938738; CNR1: FM162130; GHR: AF332042; BRCA1: AF540624; ATP7A: JF938764; Crem: JF938840; RAG2: FM162097
	vali	IRBP: AM407923; vWF: AJ238387
Aplodontia	*rufa*	A2AB: AJ427256; CNR1: AY303279; GHR: AF332030; IRBP: AF297284; BRCA1: AF332045; vWF: AJ224662; ATP7A: JF938763; Crem: JF938839; RAG2: AY303198

Ochotona	*princeps*	A2AB: AJ427253; CNR1: AY303188; GHR: JF938867; IRBP: AY057832; BRCA1: AF540635; vWF: AJ224672
	hyperborea	ATP7A: AY011409; Crem: AY011655; RAG2: AY011953
Sylvilagus	*floridanus*	A2AB: JF938737; CNR1: AY011591; GHR: AF332017; IRBP: JF938890; BRCA1: JF938789; vWF: JF938712; ATP7A: AY011408; Crem: JF938838; RAG2: AY011952
Lemur	*catta*	A2AB: AJ891067; CNR1: AY011595; GHR: JF938866; IRBP: AJ313470; BRCA1: DQ354455; vWF: AJ410292; ATP7A: AY011412; Crem: AY011658; RAG2: AY011956
Homo	*sapiens*	A2AB: NM000682; CNR1: NM001160260; GHR: NM000163; IRBP: M22453; BRCA1: NM007294; vWF: NM000552; ATP7A: NM000052; Crem: AY011664; RAG2: NM000536.2
Canis	*familiaris*	A2AB: AJ891051; CNR1: XM539034; GHR: NM001003123; IRBP: DQ205906; BRCA1: NM001013416; vWF: NM001002932; ATP7A: XM549096; Crem: AY011682; RAG2: XM540537.2
Felis	*catus*	A2AB: AJ251174; CNR1: AY011615; GHR: EU448991; IRBP: Z11811; BRCA1: AF284018; vWF: AF061062; ATP7A: AY011433; Crem: AY011679; RAG2:AY011977
Equus	*caballus*	A2AB: Y15945; CNR1: AY011612; GHR: XM001498656; IRBP: U48710; BRCA1: AF284010; vWF: XM001915467; ATP7A: AY011430; Crem: AY011676; RAG2: AF447533
Monodelphis	*domestica*	A2AB: XM001382024; CNR1: XM001362407; GHR: NM001032976; IRBP: XM001371829; BRCA1: AY994160; vWF: AY243415; ATP7A: XM001363299; Crem: ENSMODG00000006900; RAG2: XM001362253
