# Supplementary material for: Molecular and Paleontological Evidence for a Post-Cretaceous Origin of Rodents
Source: PLoS One. 2012 Oct 5;7(10):e46445. doi: 10.1371/journal.pone.0046445 (PMC3465340; doi:10.1371/journal.pone.0046445)
Supplement: Table S6 — Comparisons of divergence times for major nodes estimated using BEAST with full taxa and with a tree that is free of NDE by reducing taxon sampling in the subfamilies Dipodinae and Allactaginae. Values in parentheses are the 95% Bayesian credibility intervals. Note that these two analyses produced similar estimates of divergence times for major nodes. Statistical test shows that there is no significant difference between these two time estimates (t-test, p-value = 0.954). (PDF) [file pone.0046445.s011.pdf]

Table S6. Comparisons of divergence times for major nodes estimated using BEAST[12] with full taxa and with a tree that is free of NDE by reducing taxon sampling in the subfamilies Dipodinae and Allactaginae. Values in parentheses are the 95% Bayesian credibility intervals. Note that these two analyses produced similar estimates of divergence times for major nodes. Statistical test shows that there is no significant difference between these two time estimates (t-test, p-value = 0.954).

Node	Description of node	Full taxa	Reduced taxa
1	Marsupialia-Placentalia	165.3 (160-175.7)	166 (160-177.8)
2	Base of Boreoeutheria	69.8 (58.6-81.9)	70.6 (58.8-83.3)
3	Perissodactyla-Carnivora	56.5 (44.2-69.5)	55.4 (43.7-68.1)
4	Caniformia-Feliformia	41.6 (38-48.4)	41.5 (38-47.9)
5	Primates-Glires	65.2 (55-75.4)	66.9 (56-78.3)
6	Hominoidea-Lemuroidea	55.6 (40.4-70)	58.1 (42.9-72.4)
7	Lagomorpha-Rodentia	61.7 (52.8-71)	63.2 (53.6-73.8)
8	Ochotonidae-Leporidae	34.6 (18.8-50.7)	36.5 (19.7-52.4)
9	Base of Rodentia	57.7 (50.1-66)	58.8 (50.5-68.3)
10	Base of Hystricomorpha	50.2 (41.6-58.7)	51.2 (42.2-60.2)
11	Hystricidae-Caviomorpha	36.9 (31.6-43)	37.4 (31.8-43.7)
12	*Octodontomys-Erethizon*	29.8 (28.5-32.2)	29.9 (28.5-32.4)
13	*Cavia-Erethizon*	24.6 (17.6-30)	24.9 (18.4-30.2)
14	Myodonta-Sciuromorpha	55.6 (48.4-63.2)	56.9 (48.8-65.7)
15	Base of Sciuromorpha	25.4 (11.9-38.6)	27.3 (13.4-42.2)
16	*Glaucomys-Tamias*	17.8 (7.3-29.9)	19.5 (7.5-32.5)
17	Myodonta-Castorimorpha	52.9 (46.5-59.9)	54.3 (46.9-62.6))
18	Base of Castorimorpha	45.5 (33.9-55.3)	47 (35.5-57.5)
19	Heteromyidae-Geomyidae	20.6 (9.9-32.2)	20.8 (10.1-32.2)
20	Muroidea-Dipodoidea	45.4 (43-49.4)	45.7 (43-50.1)
21	Cricetidae-Muridae	23.4 (14.7-32.2)	23.7 (14.9-32.6)
22	Mouse-Rat	9.5 (7.3-12.9)	9.6 (7.3-13)
24	Base of Dipodoidea	32.4 (25.2-39.7)	32.7 (25.1-40)
25	Zapodidae-Dipodidae	25.3 (19.1-31.7)	25.2 (19-32.2)
26	*Zapus-Napaeozapus*	5.7 (1.9-10.1)	5.8 (2.1-10.2)
27	Base of Dipodidae	18.3 (13.9-22.8)	17.6 (13.6-22)
28	*Salpingotus-Cardiocranius*	10.2 (9-12.4)	10.2 (9-12.2)
29	Euchoreutinae-Allactaginae	14.1 (11.2-17.3)	13 (10.9-15.5)
30	Dipodinae-Allactaginae	12.4 (10.5-14.9)	11.6 (10.5-13.4)
31	Base of Allactaginae	7.7 (5.4-9.9)	5.8 (3.1-8.6)
37	Base of Dipodinae	7.5 (5-9.8)	6 (3.4-8.6)
